# Hypothyroidism associated with short bowel syndrome in children: a report of six cases

**DOI:** 10.20945/2359-3997000000093

**Published:** 2018-10-01

**Authors:** Ananda Castro Vieira Passos, Fábio de Barros, Durval Damiani, Beatriz Semer, Wendy Cira Justiniano Cespedes, Bruna Sannicola, Ana Cristina Aoun Tannuri, Uenis Tannuri

**Affiliations:** 1 Universidade de São Paulo Universidade de São Paulo Faculdade de Medicina Laboratório de Investigação em Cirurgia Pediátrica São Paulo SP Brasil Serviço de Cirurgia Pediátrica e Transplante Hepático, Laboratório de Investigação em Cirurgia Pediátrica (LIM-30), Faculdade de Medicina da Universidade de São Paulo (FMUSP), São Paulo, SP, Brasil; 2 Universidade de São Paulo Universidade de São Paulo Faculdade de Medicina Unidade de Endocrinología Pediátrica São Paulo SP Brasil Unidade de Endocrinología Pediátrica, Divisão de Pediatria, Faculdade de Medicina da Universidade de São Paulo (FMUSP), São Paulo, SP Brasil

## Abstract

Short bowel syndrome (SBS) is the leading cause of intestinal failure in children, a condition of absence of sufficient bowel to meet the nutritional and metabolic needs of a growing individual. The treatment of patients in this situation is based on the association of parenteral and enteral nutrition for prolonged periods of time until intestinal rehabilitation occurs with complete enteral nutrition autonomy. Six consecutive cases of children with SBS (residual intestinal length of 5 cm to 75 cm) were managed with this program and were diagnosed with associated hypothyroidism during the treatment (ages at the diagnosis 5 months to 12 years). All patients were successfully treated with oral hormone reposition therapy and in one patient, the replacement was performed via rectal enemas due to a complete absence of small bowel. Although iodine deficiency associated to long-term parenteral nutrition is a well-known condition, this is the first report in the literature about an expressive number of patients with hypothyroidism detected in patients with SBS during the prolonged treatment for intestinal rehabilitation.

## INTRODUCTION

Intestinal failure in children occurs in the absence of sufficient bowel to meet the nutritional and metabolic needs of a growing and developing individual (1-4). Short bowel syndrome (SBS) is the leading cause of intestinal failure in children and is characterized by a small intestine length 25% shorter than expected for gestational age (5-7). The main causes of SBS in children are massive resections secondary to necrotizing enterocolitis, gastroschisis, intestinal atresia and intestinal volvulus (1-5,8).

SBS-associated intestinal failure causes various metabolic changes in the body, many of which are associated with growth, intestinal adaptation and bone metabolic disease. Research on SBS and growth hormone (GH), glucagon-like peptide-2 (GLP-2), vitamin D and parathyroid hormone (PTH) is already well advanced; in contrast, little is known about the hypothalamic-pituitary-thyroid (HPT) axis and its balance in patients with SBS (3,8,9).

In critically ill patients with acute or chronic disease, decreased serum triiodothyronine (T3) and thyroxine (T4) levels are associated with normal or reduced concentrations of thyroid stimulating hormone (TSH). This set of findings is known as nonthyroidal illness syndrome (NTIS) (10-14). Periods of caloric restriction were also associated with low concentrations of T3 and T4 without increased TSH, suggesting that central action mechanisms are also responsible for inhibition of the HPT axis (11,12,15-17).

It is not known whether these changes are part of a protective mechanism for decreasing basal metabolism and saving energy during critical periods or if they are part of an erroneous response to insult, motivated by the presence of inflammatory cytokines. Thus, accurate indications of the correction of these hormonal changes have not yet been fully established (10-12). In turn, the correct functioning of the HPT axis favors the intestinal adaptation process, and the replacement of thyroid hormones in experimental models of SBS has shown benefits in intestinal adaptive parameters (i.e., enterocyte proliferation and increased crypt depth and villi height) (18). Finally, in addition to the thyroid changes caused by metabolic changes intrinsic to SBS, there is also the possibility of imbalance in the HPT axis secondary to iodine deficiency (19).

HPT axis deficiency may lead to clinical hypothyroidism, which is classified as primary if the defect is at the thyroid gland, secondary if the problem is the TSH production by the hypophysis and tertiary if the hypothalamus does not produce thyrotropin releasing hormone (TRH) (13).

The cases of six patients with SBS who presented changes in the HPT axis and hypothyroidism, during treatment of intestinal failure at the Children's Institute of the University of São Paulo School of Medicine, from 2012 to 2017, are reported herein. As far as we know, this is the first report of an expressive number of patients with such association although iodine deficiency associated to long-term parenteral nutrition is a well-known condition and may be avoided by adequate intravenous administration of sodium iodine (20).

## REPORTS

The medical records of 25 patients with short bowel syndrome treated at the Unit's intestinal rehabilitation program were reviewed, of whom six presented with hypothyroidism (Table 1). It is important to note that all laboratory tests were repeated in order to adequately confirm the alterations.

**Table 1:** Summary of clinical data of SBS patients with hypothyroidism

Patient/age at SBS	Residual small bowel (cm)	Hypothyroidism cause	Nutritional status*	Daily calories Parenteral/ enteral (kcal/ kg/day)	Age at hypothyroidism	TSH** (μlU/mL)	T3/T4** (ng/dL)/ (μg/dL)	Free T4** (ng/dL)
1/new born	75	NTIS	Undernourished	70/75	3 years	3.9	51/4.7	0.7
2/3 months	35	NTIS	Undernourished	40/130	7 months	2.3	- /3.4	< 0.3
3/new born	40	Iodine deficiency	Eutrophic	60/70	5 months	10.6	137/7.0	0.7
4/new born	50	Iodine deficiency	Eutrophic	- /200	3 years	19.8	110/8.1	1.5
5/8 years	15	Iodine deficiency	Eutrophic	50/ -	12 years	13.1	86/-	0.8
6/5 years	5	Iodine deficiency	Eutrophic	45/ -	11 years	> 300.0	< 47/< 0.8	< 0.3

* Nutritional status at the time of hypothyroidism diagnosis.

** Exams at the time of hypothyroidism diagnosis.

### Case 1

RGPC, 4 years old, SBS secondary to gastroschisis and intestinal resection, with 75 cm residual small bowel, dependent on parenteral nutrition (PN) since birth, is currently been weaned from PN. Currently, the patient is at the 5 th percentile for weight and height. Screening tests for thyroid function showed normal serum concentrations of TSH (3.95 μlU/mL; normal range 0.27 to 4.20 μlU/mL) and low serum concentrations of T3 (51 ng/dL; reference value [RV]: 80 to 200 ng/dL), T4 (4.7 μg/dL; RV: 5.1 to 14.1 μg/dL) and free T4 (0.73 ng/dL; RV: 0.93 to 1.70 ng/dL). In addition, pituitary and hypothalamic alterations were ruled out by laboratory tests (the serum levels of gonadotrophic hormones, growth hormone, adrenocorticotrophic hormone were normal, and magnetic resonance image was normal). After five months of hormone replacement therapy the levels of T3, T4 and free T4 (FT4) became normal, without significant clinical changes.

### Case 2

PGFSV, 8 months old, SBS secondary to volvulus, with 35 cm of residual small bowel, dependent on PN since the time of intestinal resection performed at 3 months of age. Laboratory tests showed normal TSH (2.26 μlU/ mL; RV: 0.27 to 4.2 μlU/mL) and very low T4 and FT4 levels (3.4 μg/dL and < 0.3 ng/dL respectively). The laboratory tests and resonance image ruled out pituitary and hypothalamic alterations. At this time, the patient presented with severe malnutrition, with weight and height below the 5^th^ percentile, hypoactivity and hyporeflexia. After 1 month of hormone replacement therapy, the patient's T4 and FT4 levels returned to normal values, and the patient improved clinically, with weight gain and normal reactivity.

### Case 3

PHLO, 4 years old, SBS secondary to gastroschisis, with 40 cm of residual small bowel, dependent on PN since the neonatal period until 2 years of age, is currently weaned from PN, and he is in the 50th percentile of weight and 25th percentile of height. The patient was diagnosed with primary hypothyroidism at 5 months of age (TSH 10.56 μlU/mL, T3 137 ng/dL, T4 7.0 μg/dL, FT4 0.73 ng/dL). The investigation for the presence of thyroid autoantibodies was negative. Administration of exogenous thyroid hormone for 1 year and 10 months promoted normalization of serum hormone concentrations. Subjectively, both the family and the healthcare team noticed a decrease in the volume and an increase in the consistency of bowel movements, with no other significant clinical changes after initiation of therapy. Currently, the patient does not receive levothyroxine (L-T4) and remains euthyroid.

### Case 4

NVSM, 4 years old, SBS secondary to gastroschisis, with 50 cm of residual small bowel, PN dependent until 3 years of age, is not currently receiving PN, and he is maintaining stable nutritional status in the 10^th^ percentile of weight and 2.5^th^ percentile of height. Thyroid screening tests performed at 4 months of age showed no abnormalities. Subsequent tests at 3 years of age diagnosed primary hypothyroidism (TSH 19.79 μlU/mL, T3 110 ng/dL, T4 8.1 μg/dL, FT4 1.48 ng/ dL). Negative thyroid autoantibodies. The patient still receives hormone replacement therapy started at 3 years of age. Despite the improvement in laboratory tests, the administration of L-T4 did not cause significant clinical changes. Currently, the child feeds exclusively by oral route and has normal bowel movements.

### Case 5

GHMM, 14 years old, SBS secondary to massive resection due to necrosis secondary to intestinal volvulus at 8 years of age. The patient currently has 15 cm of small intestine anastomosed to the transverse colon. The patient is dependent on PN daily and ingests minimal amounts of food orally. The patient remains eutrophic, in the 50^th^ percentile of weight and height. At the age 12, during routine testing, the patient was diagnosed with primary hypothyroidism (TSH 13.14 μlU/mL, T3 86 ng/dL, 0.82). The thyroid autoantibodies were absent. The patient had no symptoms of hypothyroidism at the time of diagnosis. Oral administration of L-T4 normalized serum thyroid hormone concentrations although no evident clinical changes were noted.

### Case 6

VTR, 11 years old, SBS caused by massive intestinal necrosis after bowel volvulus at 5 years of age. The patient has only part of duodenum, which is exteriorized via an ostomy in the mesogastrium, and 5 cm of rectum, which is buried in the pelvis. He was referred to our Service in bad conditions and undernourished (body weigh – 13 kg). The patient is totally PN-dependent and does not intake any food or medication orally. Despite that, he has been remained eutrophic, in the 50th percentile of weight and height. The clinical evaluations after 6 years of PN treatment showed excessive weight gain, hypoactivity, hyporeflexia and increased cervical thyroid gland volume (current body weight – 49kg). The laboratory tests showed that TSH was above 300 μlU/mL, T3 < 47 ng/dL, T4 < 0.8 μg/dL) and FT4 < 0.3 ng/dL. The thyroid autoantibodies were absent. Cervical ultrasound showed a diffusely enlarged thyroid with no nodules. Subsequent investigation indicated primary hypothyroidism due to iodine deficiency (due to problems involving pharmacological incompatibility with other trace elements, iodine was not included in PN solutions). L-T4 hormone replacement via rectal enemas normalized the patient's thyroid hormone levels, and the patient presented significant clinical improvement, including weight loss.

Finally, the data of these six patients were compared to the other 19 euthyroid patients with SBS cohort (Table 2).

**Table 2:** Data comparing all SBS patients with respect to thyroid function and nutritional treatment

Cases	Central hypothyroidism (NTIS - cases 1 and 2)	Iodine deficiency (cases 3, 4, 5, and 6)	Euthyroidism (19 patients)
Age	8m – 4y	4y – 14y	1y – 7y
Age at SBS diagnosis	0m – 3m	0m – 8y	0m – 4m
SBS cause	Gastroschisis/volvulus	Gastroschisis/volvulus	Gastroschisis/volvulus/necrotizing enterocolitis/intestinal atresia
Residual small bowel (cm)	35 – 75	5 – 50	5 – 50
Age at hypothyroidism	7m – 3y	5m – 12y	No
Parenteral nutrition calories (%)	26 – 50	0 – 100	0 – 80
Nutritional status	Undernourished	Eutrophic	Undernourished/Eutrophic
Iodine supplementation	No	No	No
T3 (ng/dL)	51.0	<47.0 – 137.0	82.0 – 179.0
T4 (μg/dL)	3.4 – 4.7	<0.8 – 81.0	5.7 – 11.3
Free T4 (ng/dL)	2.26 – 3.95/< 0.3 – 0.73	10.53 - >300 / <0.3 – 1.48	0.98 – 1.62
TSH (μlU/mL)	2.26 – 3.95	10.53 - >300	0.67 – 4.03

Normal Range: TSH 0.27 to 4.20 μlU/mL; T3 80 to 200 ng/dL; T4 5.1 to 14.1 μg/dL; Free T4 0.93 to 1.70 ng/dL.

## DISCUSSION

In the last century, there was only one reference in the literature reporting the occurrence of hypothyroidism in two pediatric patients with SBS under parenteral nutrition therapy (21). However, with the widespread prolonged use of this therapy in children and adults, few other publications have been showing such complication in the current century (22,23). We report here the occurrence of hypothyroidism observed in an expressive number of patients with SBS, during the period of intestinal rehabilitation. Considering the great number of patients with SBS, both children and adults, that have been submitted to prolonged periods of parenteral nutrition for the intestinal rehabilitation, we conclude the importance of the present publication. In fact, it is the sequence of a previous repor from our Service about the intestinal rehabilitation program for the treatment of children with SBS (20).

Regarding iodine metabolism and parenteral nutrition therapy, an important historical aspect may be stressed. Since 20 or 25 years ago, the widespread practice of utilizing iodine-based antiseptics has been abandoned, although it prevented the patients to have iodine deficiency, even though this element was not administered. Certainly, the abandon of iodine-based formulas to 2% chlorhexidine solutions for skin and catheter hygiene may have caused iodine deficiency and hypothyroidism when iodine is not adequately provided to the patient (24). Similar to other centers, our parenteral nutrition solutions do not contain iodine, and supplies by oral route higher than the classical amount of 1 mg/kg/day may be considered in all children with any remaining intestine (25-28). Finally, in cases with relevant clinical symptoms of hypothyroidism, besides the iodine administration, the L-T4 hormone replacement may be advocated.

The cases reported herein refer to children with the same underlying disease but with different hormonal axis changes. In the first two cases, the thyroid function findings were similar to those found in children with NTIS that is a condition more frequent than pituitary or hypothalamic related diseases. Also, it has already been described in patients with severe baseline pathologies (acute or chronic), and, to date, can be explained by increases in inflammatory cytokines, such as TNF-α, which block the activity of 5'-deiodinase (responsible for the conversion of T4 to T3) and other central mediators that reduce the release of TSH (10-13). Laboratory findings similar to those of NTIS are also observed in experimental models of caloric restriction (acute or chronic) (12,15-17,29). There are no experimental studies that specifically relate SBS to the HPT axis; however, since SBS is a serious condition involving caloric restriction, we can infer that the mechanisms involved in thyroid changes in SBS are the same as those involved in NTIS and in experimental models of chronic caloric restriction. These two patients were treated with hormone replacement, and their T3, T4 and FT4 concentrations normalized. The first patient had no clinical changes after treatment and, despite having the longest residual bowel (75 cm), did not present significant intestinal adaptation and was still dependent on PN. The other patient presented significant clinical improvement, with reversal of hypoactivity and weight gain. However, this clinical response coincided with increased enteral and parenteral caloric intake and consequent improvement in the patient's nutritional status. Therefore, it was difficult to conclude that hormone replacement was, in fact, beneficial or whether clinical improvement would occur regardless of L-T4 administration. Theoretically, the decrease in the basal metabolism caused by the downregulation of the HPT axis would be a protection mechanism during critical periods. In this sense, hormone replacement is debatable (10-17,29,30). Further studies are needed to define the actual role of L-T4 administration in these situations, especially in SBS, since thyroid hormones not only modulate basal metabolism but also have a trophic effect on the intestine, a fact that is especially important for children in the intestinal adaptation phase (18).

The other four cases refer to patients with a diagnosis of primary hypothyroidism, with initial levels of TSH greater than 10 μlU/mL and low FT4 or FT4 at the lower limit of normal. The main causes of primary hypothyroidism are iodine deficiency, autoimmune thyroiditis, cervical radiotherapy and radioiodine therapy. These patients were negative for autoimmune thyroiditis, and none of them had undergone radiotherapy or radioiodine therapy. The cause of primary hypothyroidism in patients with SBS is likely multifactorial, and iodine deficiency (either due to low intake or non-absorption) plays a key role in this process. The iodine contained in ingested foods is absorbed in the small bowel (31) and is incorporated into the tyrosine residues of thyroglobulin molecules (13). It is known that minimal daily amounts of iodine (1 μg/kg) are sufficient to maintain the body's reserve (32). Patients with SBS have insufficient small bowel length and do not absorb adequate amounts of micronutrients and should therefore receive them parenterally.

It is interesting to comment that one of the patients (case 3), after achieving intestinal autonomy and becoming independent of PN, no longer needed hormone replacement. This case reinforces the idea of the relationship between intestinal failure and euthyroid status. In contrast, in another patient (case 4), this relationship was not evidenced, since despite the fact that the child is no longer dependent on PN to maintain stable growth and development, he still needs to receive L-T4. It is important to note that the other 19 children with SBS treated in our program of intestinal rehabilitation, with intestinal lengths similar to those reported herein, have no clinical hypothyroidism and have normal thyroid laboratory tests. The reason why children with similar intestinal lengths receiving similar amounts of nutritional and iodine intake manifest (or not) thyroid disease is not well established and requires further study.

The last two cases are patients with SBS secondary to bowel volvulus. They fed normally until the age at which the volvulus occurred; therefore, they had normal iodine reserves. Both patients currently receive virtually no oral feeding and, consequently, negligible amounts of enteral iodine. Antisepsis of the devices of these patients is not performed with iodinated substances. One of them (case 6) developed clinical hypothyroidism 5 years after volvulus, and the other (case 5) was diagnosed with hypothyroidism (through laboratory investigation) 4 years after volvulus. There have been few studies on the time needed to fully deplete iodine reserves, with some reporting that depletion would occur within a few months (19).

Finally, one patient (case 6) presented an emblematic picture of clinical hypothyroidism secondary to a lack of iodine in parenteral nutrition administered solutions. This patient cannot intake no food orally, and even if he does, the whole food bolus would exit via the duodenostomy located 5 cm from the pylorus. The almost complete absence of intestinal absorption surface in this patient made it difficult to control their hypothyroidism. The administration of enteral iodine was not effective, and the administration of L-T4 orally also did not alter the serum concentrations of TSH and FT4. It is noticeable that the patient is currently receiving L-T4 rectally and has normal concentrations of thyroid hormones. This route of administration of the exogenous hormone has been previously described (33).

In conclusion, changes in the HPT axis associated with SBS are multifactorial and involve other metabolic reactions that have not yet been fully elucidated. When the child becomes dependent on total parenteral nutrition and does not present conditions for oral food intake, iodine deficiency eventually leads to a primary hypothyroidism, condition similar to endemic goiter, and L-T4 replacement normalizes the clinical picture. In SBS cases where iodine deficiency is not as evident (i.e., partial enteral feeding), further studies are needed to define the complex relationships between SBS and the HPT axis and to guide therapy. It is important that the thyroid hormone levels be periodically evaluated in every child with SBS and included in a program of intestinal rehabilitation.
